# 355 nm Nanosecond Ultraviolet Pulsed Laser Annealing Effects on Amorphous In-Ga-ZnO Thin Film Transistors

**DOI:** 10.3390/mi15010103

**Published:** 2024-01-05

**Authors:** Sang Yeon Park, Younggon Choi, Yong Hyeok Seo, Hojun Kim, Dong Hyun Lee, Phuoc Loc Truong, Yongmin Jeon, Hocheon Yoo, Sang Jik Kwon, Daeho Lee, Eou-Sik Cho

**Affiliations:** 1 Department of Electronics Engineering, Gachon University, Seongnam City 13120, Republic of Korea; tkdus9@gachon.ac.kr (S.Y.P.); cyg1994@gachon.ac.kr (Y.C.); danny99hodam@gachon.ac.kr (D.H.L.); hyoo@gachon.ac.kr (H.Y.); sjkwon@gachon.ac.kr (S.J.K.); 2 Department of Mechanical Engineering, Gachon University, Seongnam City 13120, Republic of Korea; fahenz@gachon.ac.kr (H.K.); 202050078@gachon.ac.kr (P.L.T.); 3 Department of Biomedical Engineering, Gachon University, Seongnam City 13120, Republic of Korea; yongmin@gachon.ac.kr

**Keywords:** UV pulsed laser annealing, a-IGZO TFT, ITO/IGZO energy band structure, selective annealing

## Abstract

Bottom-gate thin-film transistors (TFTs) with n-type amorphous indium-gallium-zinc oxide (a-IGZO) active channels and indium-tin oxide (ITO) source/drain electrodes were fabricated. Then, an ultraviolet (UV) nanosecond pulsed laser with a wavelength of 355 nm was scanned to locally anneal the active channel at various laser powers. After laser annealing, negative shifts in the threshold voltages and enhanced on-currents were observed at laser powers ranging from 54 to 120 mW. The energy band gap and work function of a-IGZO extracted from the transmittance and ultraviolet photoelectron spectroscopy (UPS) measurement data confirm that different energy band structures for the ITO electrode/a-IGZO channel were established depending on the laser annealing conditions. Based on these observations, the electron injection mechanism from ITO electrodes to a-IGZO channels was analyzed. The results show that the selective laser annealing process can improve the electrical performance of the a-IGZO TFTs without any thermal damage to the substrate.

## 1. Introduction

Metal-oxide semiconductors have garnered significant interest as core materials for next-generation displays, primarily owing to their low off-current and suitability for low-temperature processing. In particular, they can be deposited through low-temperature sputtering, a method that enhances process compatibility, leading to improved uniformity across large areas and heightened reliability over extended periods, as evidenced in several studies [1,2,3,4,5,6]. Amorphous indium-gallium-zinc oxide (a-IGZO) has been widely used as a channel material for thin-film transistors (TFTs) because of its moderate electron affinity, low threshold voltage, and high transmittance caused by its high-energy band gap [7,8,9,10,11,12]. Although a-IGZO has a relatively high energy bandgap, the orbital overlap of indium and oxygen vacancies in the zinc oxide (ZnO) structure enables it to possess high electron mobility and electrical conductivity [13,14,15]. The ionic bonding of a-IGZO maintains high carrier mobility in amorphous metal-oxide semiconductors, in contrast to the covalent bonding of amorphous Si. At a positive gate voltage (on condition), an a-IGZO TFT can show 20–50 times higher electrical current than a hydrogenated amorphous silicon (a-Si:H) TFT, and the off-leakage current of an a-IGZO TFT is much lower than that of a low-temperature polysilicon (LTPS) TFT [16]. Therefore, a higher speed without motion blur and low power consumption can be obtained while driving display devices.

In previous studies, a-IGZO thin films were thermally annealed using an oven, a vacuum furnace, or rapid thermal annealing (RTA) to improve the electrical stability and reliability. Considering the melting point of the soda-lime glass substrate, the annealing temperatures have been maintained under 500–600 °C [17,18,19,20]. However, the completion of the annealing processes requires a relatively long time because of the cooling process time in RTA and the stabilization time in a vacuum furnace. Moreover, the high temperatures involved in conventional annealing are not compatible with flexible substrates such as polyimide (PI) or polyethylene terephthalate (PET). As a new low-temperature rapid annealing process, a xenon (Xe) flash lamp for the annealing of a-IGZO TFT was applied in a previous study, which results in enhanced electrical characteristics of the TFT [21]. However, since the annealing process using a Xe lamp is a process applied to the entire substrate, unintended side effects, such as electrode damage, can occur [22].

For the selective annealing process, a laser was used in the fabrication of a-Si:H TFTs, and LTPS TFTs were used as driving TFTs in active-matrix organic light-emitting diode (AMOLED) displays. This process, however, has some drawbacks, such as the high cost of laser equipment and damage to thin films caused by laser beam scanning [23,24]. Because lasers induce localized heating in a very short time during the annealing process, no specific cooling is required, thus the process can be completed in a short time. Furthermore, the digitized laser parameters, such as pulse duration, repetition rate, and scanning speed, can be easily controlled and optimized [25,26,27].

In this study, we irradiated active layers of a-IGZO TFTs using a 355 nm ultraviolet (UV) nanosecond pulsed laser to investigate and analyze the annealing effects on the electrical characteristics of the TFT. Considering the TFT size used in the display industry, a-IGZO TFTs with channel lengths of less than 10 μm were fabricated. The nanosecond pulsed laser was scanned to locally anneal the active channels at various laser powers up to 280 mW. The electrical characteristics of the TFTs depending on the laser annealing powers were analyzed. To investigate the structural characteristics of the laser-annealed a-IGZO TFTs, a-IGZO thin films were deposited on glass and Si wafer substrates, and the laser was irradiated on the thin films under identical conditions. Table 1 shows a comparison chart that distinguishes between the laser annealing process for a-IGZO used in this study and previous studies on laser annealing for a-IGZO or similar oxide TFTs.

## 2. Experimental Methods

Figure S1 shows the sequence of the fabrication process of the a-IGZO/ITO TFT. A boron-doped p-type silicon wafer with a resistivity of 1–10 Ω cm was used as a substrate. For the gate dielectric layer of the TFT, silicon dioxide (SiO_2_) was thermally grown on the Si substrate. Before sputtering of ITO for source/drain electrodes, the silicon wafer was cleaned with isopropyl alcohol (IPA), acetone, and deionized (DI) water in an ultrasonic bath for 15 min. The cleaned silicon wafer was vertically loaded into a jig in the load-lock chamber, and moved to an in-line sputter chamber. The silicon substrate was positioned in front of a 99.99% indium tin oxide (ITO) target (InO_3_:SnO_2_ = 90:10), measuring 165 mm × 540 mm × 7 mm, at a base vacuum of 9 × 10^−6^ Torr. Plasma was generated using a DC power of 2 kW at a pressure of 4 mTorr at room temperature. Gas injections of Ar and O_2_ were maintained at rates of 50 and 1.2 sccm, respectively [31,32]. During ITO sputtering, the jig was scanned at a speed of 45 cm/min (15 Hz) in front of the ITO target. The thickness and sheet resistance of the ITO target were about 80 nm and 65.5 ± 0.6 Ω/sq, respectively.

In the photolithography process for the ITO electrode, AZ GXR 601 positive photoresist (PR) was spin-coated at 4000 rpm. The PR underwent a soft bake on a hot plate at 90 °C for 60 s, followed by UV irradiation for 3 s through Cr patterns for source and drain electrodes on the photomask. The distance between the mask and photo mask was maintained at 100 μm. PR development was carried out using AZ 300 MIF developer solution after post-exposure baking on a hot plate at 110 °C for 60 s. The PR was then hard-baked, and the ITO source and drain patterns were obtained by wet etching using an AP-KIT ITO etchant at room temperature for 20–30 s, as shown in Figure S1A. For the IGZO active layer patterning, the same photolithography process parameters were applied as with the ITO patterning, as shown in Figure S1B. Figure S1C shows the a-IGZO active layer deposited to a thickness of 30 nm using radio frequency (RF) sputtering at room temperature for 210 s. An IGZO target (In_2_O_3_:Ga_2_O_3_:ZnO = 1:1:1) measuring 4 inches in diameter and 1/8 inch in thickness was used and plasma was generated with an RF power of 60 W at a pressure of 4 mTorr. The lift-off process was performed using acetone dipping and N_2_ blowing, resulting in the formation of an IGZO/ITO TFT, as shown in Figure S1D.

**Figure 1:**
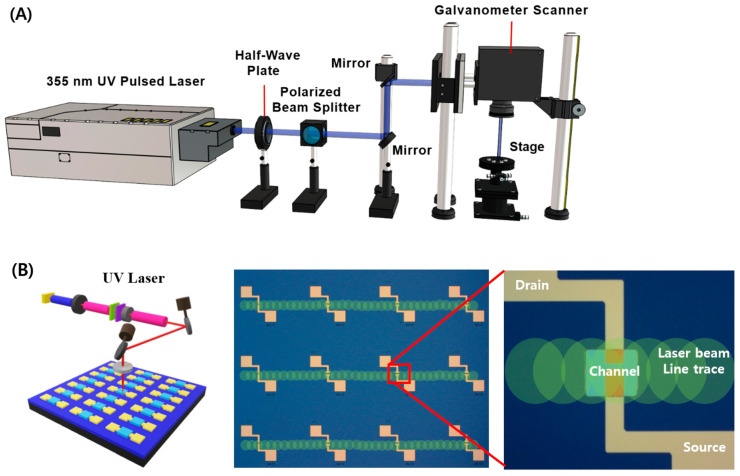
Schematic of (**A**) a laser annealing system using a 355 nm UV nanosecond pulsed laser and (**B**) the experimental procedure for laser annealing on a-IGZO TFTs. Multiple TFTs were scanned using the laser and the scanning direction of laser beam was perpendicular to the direction of the TFT channel.

Figure 1A shows the laser system setup used for the laser annealing of the a-IGZO/ITO TFT. A 355 nm diode-pumped solid-state Nd:YVO_4_ pulsed laser (Poplar 355-3A, Huaray, Wuhan, China) was used. The pulse repetition rate and pulse duration of the laser were 100 kHz and 35 ns, respectively, and the laser beam diameter focused through a telecentric lens equipped in the galvanometer scanner was 20 μm. The laser power was controlled precisely by rotating the half-wave plate installed in front of the polarized beam splitter. The laser beam was scanned across the a-IGZO channel layers of a-IGZO/ITO TFTs. The laser-beam scanning speed was maintained at 100 mm/s, and the scanning direction was aligned for application to multiple TFTs in a single stroke, as shown in Figure 1B. Laser annealing was performed at laser powers ranging from 0 (no annealing) to 280 mW.

The current-voltage characteristics of the laser-annealed a-IGZO/ITO TFT were measured using a probe station and parameter analyzer (HP-4156 C). The laser scanning traces were investigated by field emission scanning electron microscopy (Hitachi S-4700). The transmittance and absorbance of the laser-annealed a-IGZO were measured using a UV visible spectrometer (VARIAN, Cary 100). To analyze the energy band structure of the laser-annealed a-IGZO, the cutoff energy and valence band maximum (VBM) were obtained using ultraviolet photoelectron spectroscopy (UPS) (ESCALAB 250, Thermo Fisher Scientific, Waltham, MA, USA).

## 3. Results and Discussion

### 3.1. Current-Voltage Characteristics Depending on Laser Annealing Powers

**Figure 2:**
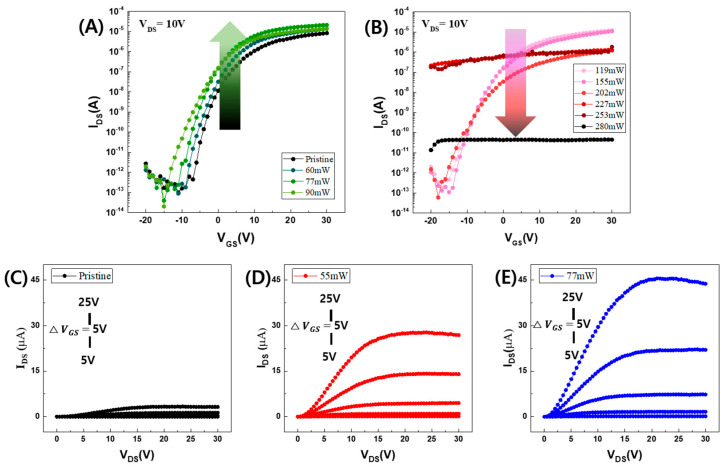
Transfer characteristics of a-IGZO TFTs after a 355 nm UV nanosecond pulsed laser annealing process (**A**) from pristine (no annealing) to 90 mW and (**B**) from 119 mW to 280 mW. Output characteristics of a-IGZO TFTs after the laser annealing process (**C**) pristine, (**D**) 55 mW, and (**E**) 77 mW.

The electrical characteristics of a-IGZO TFTs, with varying channel widths and lengths, were normalized to facilitate comparison of their transfer and output characteristics post-laser annealing. Figure 2A shows the transfer characteristics of the a-IGZO TFTs after laser annealing at various laser powers ranging from pristine (no annealing) to 90 mW. As the laser power increased to 90 mW, the threshold voltage (V_TH_) shifted in the negative direction, and the on/off current ratio increased. The subthreshold swing (S.S.) was also reduced slightly from 2.0 V/dec to 1.66 V/dec, as detailed in Table 2. However, for higher laser powers ranging from 119 to 280 mW, the electrical parameters were steeply degraded, as shown in Figure 1B. Furthermore, above 200 mW, no transfer characteristics were observed and no device parameters were obtained, as shown in Table 2. Figure 2C–E shows the output characteristics of the a-IGZO TFTs at laser annealing powers of 0 (pristine), 55, and 90 mW, respectively. An increase in the gate-to-source voltage (V_GS_) from 5 to 25 V led to a significant rise in the drain-to-source current, especially at laser powers of 55 and 77 mW. This enhanced on-current is attributed to the effects of laser annealing. 

**Figure 3:**
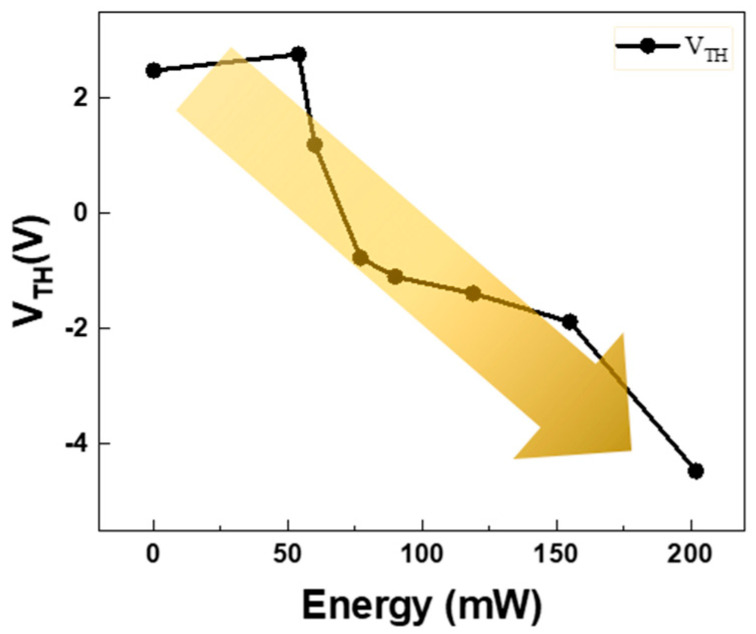
Threshold voltages (V_TH_) of a-IGZO TFTs after a 355 nm UV pulsed laser annealing process for laser powers ranging from pristine (no annealing) to 280 mW.

Table 2 also shows the dependence of the device parameters such as V_TH_, S.S., saturation mobility, and on/off current ratio (I_on_/I_off_) on the laser annealing powers. As shown in Figure 2B, when the laser power was higher than 200 mW, a consistent off-current was generated, independent of the V_GS_ value. This phenomenon was similarly observed in 308 nm XeCl excimer laser annealing, 248 nm KrF excimer laser annealing, and 800 nm femtosecond laser annealing of sputtered IGZO TFTs, as reported in references [26,28,29]. The unstable electrical characteristics are expected to be attributed to device damage caused by excessive laser power and the generation of excessive carriers during laser annealing. Notably, smaller-sized TFTs tend to degrade more under laser annealing, as inferred from two-dimensional heat simulations of laser heating [31]. As the laser power was increased to 90 mW, the enhanced on-current may have affected I_on_/I_off_, and the S.S. was also slightly improved. However, no clear correlation or dependence was observed between mobility and laser power. In the case of CO_2_ laser spiking annealing (LSA) of a-IGZO TFTs, identifying an optimized LSA condition to enhance saturation mobility is challenging, as mobility improvements do not consistently correlate with LSA parameters like peak temperatures and dwell time [32]. Figure 3 shows that V_TH_ was slightly increased at 54 mW and exhibited a linear negative shift with increasing laser power. This behavior might be attributed to the limited capacity for the relaxation of structural defects. In contrast to conventional thermal annealing, the rapid timescale of laser annealing may not allow sufficient time to prevent ionization of oxygen vacancies and to compensate for laser-induced damage, as suggested in references [30,33].

### 3.2. Structural Characteristics and Energy Band Analysis Depending on Laser Annealing Powers

**Figure 4:**
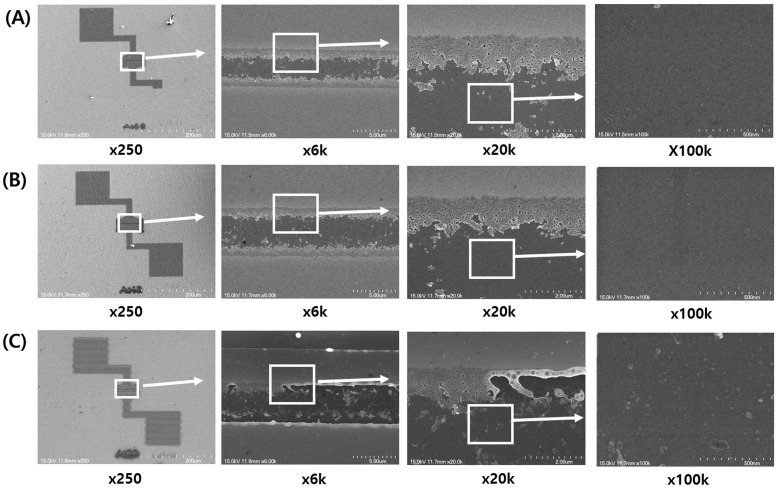
Scanning electron microscopy (SEM) images of a-IGZO TFTs after a 355 nm UV pulsed laser annealing process for the laser powers of (**A**) pristine (no annealing), (**B**) 77 mW, and (**C**) 227 mW. In case of the high energy of 227 mW, laser beam line traces are observed.

To examine the structural characteristics of the laser-annealed TFTs, Figure 4A–C show scanning electron microscopy (SEM) images of a-IGZO TFTs, laser-annealed at powers of 0 (pristine), 77 mW, and 227 mW, respectively. As shown in Figure 4B, no laser beam traces or etching residues were noticeable at a laser power of 77 mW, indicating no apparent impact of the laser power on the a-IGZO layers. However, laser line scanning traces were observed at 227 mW, as shown in the left image of Figure 4C. SEM observations of all laser-annealed TFTs disclosed laser line traces for powers exceeding 200 mW (Figure S2). These line beam traces could lead to laser-induced damage in a-IGZO TFTs, resulting in deteriorated electrical characteristics such as elevated off-currents. In the case of higher powers of 250 and 280 mW, substantial gate-source leakage currents were recorded (Figure S3), suggesting that excessive laser exposure might not only damage the a-IGZO channel layer but also affect the thermally grown SiO_2_.

**Figure 5:**
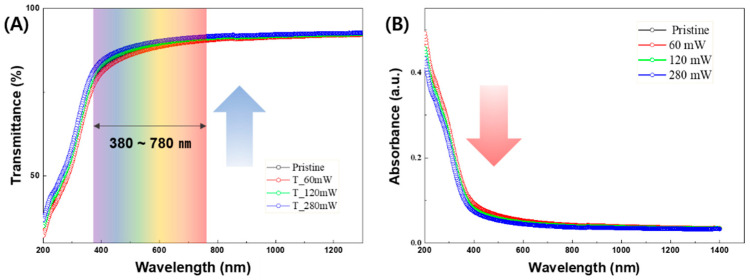
(**A**) Transmittance and (**B**) absorbance of a-IGZO thin films annealed using a 355 nm UV pulsed laser for laser powers of 0 (pristine), 60, 120, and 280 mW.

**Figure 6:**
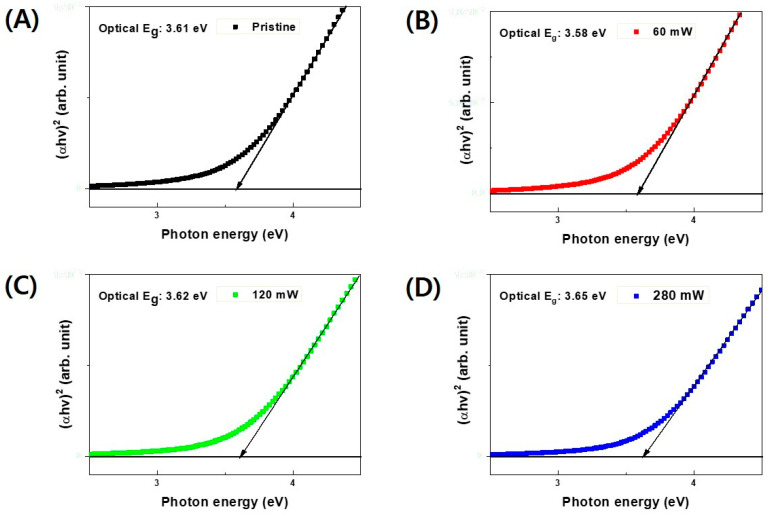
Tauc Plot for the energy band gap of a-IGZO thin films shown in Figure 5. The 355 nm UV pulsed laser powers are (**A**) 0 (pristine), (**B**) 60, (**C**) 120, and (**D**) 280 mW.

To further analyze the enhanced on-current and negative shift in V_TH_ observed in the laser-annealed a-IGZO TFTs, the optical bandgaps of the laser-annealed a-IGZO layers were obtained by measuring their transmittance data. Figure 5A,B displays the transmittance data of the laser-annealed a-IGZO thin films and their corresponding absorbance, calculated using the logarithm of the reciprocal of the transmittance, respectively. For wavelengths exceeding 400 nm, all a-IGZO thin films exhibited transmittances higher than 80%. The average transmittances ranging from 380 to 780 nm were recorded as 87.54%, 87.18%, 88.45%, and 89.22% for laser powers of 0 (pristine), 60, 120, and 280 mW, respectively, indicating a slight increase in transmittance with rising laser power. Utilizing the absorbance data from Figure 5B and the known thicknesses of the a-IGZO films, the optical bandgaps of the laser-annealed layers were calculated using a Tauc plot, as presented in Figure 6. The determined optical bandgaps of a-IGZO were 3.61 eV, 3.58 eV, 3.62 eV, and 3.65 eV for laser powers of 0 (pristine), 60 mW, 120 mW, and 280 mW, respectively.

**Figure 7:**
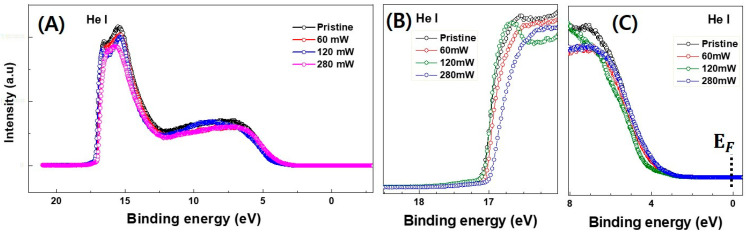
(**A**) Ultraviolet photoelectron spectroscopy (UPS) spectrum of 355 nm UV pulsed laser annealed a-IGZO thin films for direct determination of (**B**) cutoff energy and (**C**) valence band maximum (VBM).

To determine the energy band structures of the laser-annealed a-IGZO layers, UPS spectra were measured for the laser-annealed a-IGZO thin films on silicon substrates, as shown in Figure 7A. The conditions for RF sputtering and laser annealing were consistent with those described in Figure 5 and Figure 6. The cutoff energy and VBMs were derived from the detailed views in Figure 7B,C, respectively [34]. 

**Figure 8:**
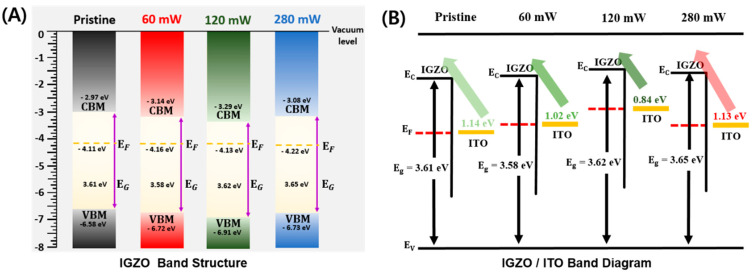
Energy band diagrams of (**A**) a-IGZO thin films and (**B**) a-IGZO/ITO junction structures obtained from the UPS results shown in Figure 7.

Figure 8A shows the energy band diagrams of the laser-annealed a-IGZO thin films. These diagrams were constructed using the work functions calculated from Figure 7B and the VBMs obtained from Figure 7C. The work functions, measured from the vacuum energy level, were found to be 4.11 eV, 4.16 eV, 4.13 eV, and 4.22 eV for laser powers of 0 (pristine), 60 mW, 120 mW, and 280 mW, respectively. The VBM values, also measured from the vacuum level, were determined to be 6.58 eV, 6.72 eV, 6.91 eV, and 6.78 eV for the same respective laser powers. Considering the optical energy bandgaps shown in Figure 6, the conduction band minimum (CBM) was calculated to be 2.97 eV, 3.14 eV, 3.29 eV, and 3.08 eV from the vacuum energy level for laser powers of 0 (pristine), 60, 120, and 280 mW, respectively.

The junction between the ITO electrode and a-IGZO channel layers was described for different laser powers, as shown in Figure 8B. Assuming that the Fermi energy level of ITO is identical to that of a-IGZO, the energy barriers for electrons to move from ITO to the a-IGZO conduction band were calculated. These barriers were found to be 1.14 eV, 1.02 eV, 0.84 eV, and 1.13 eV for laser energies of 0 (pristine), 60 mW, 120 mW, and 280 mW, respectively. Therefore, the laser power of 120 mW was identified as the optimal level, as it minimized the distance between the Fermi level and the conduction band of a-IGZO. Consequently, this reduction in the energy barrier between the ITO electrode and the channel led to an increase in carrier concentration and on-current. 

## 4. Conclusions

We investigated the effects of 355 nm UV nanosecond pulsed laser annealing on the performance of a-IGZO TFTs. The laser beam was scanned to locally anneal the a-IGZO active channels at various laser powers. After laser annealing, negative shifts in the threshold voltages and enhanced on-currents were observed at laser powers ranging from 54 to 120 mW. UPS analysis revealed that the energy barrier between the CBM and Fermi level decreased after laser annealing, resulting in an increase in the carrier concentration at the surface. SEM analysis confirmed that no thermal damage occurred on the substrate during the annealing process with laser powers lower than 120 mW. The results show that the selective laser annealing process can improve the electrical performance of the a-IGZO TFTs effectively.

## Figures and Tables

**Table 1:** Comparison chart of laser annealing methods for oxide TFTs.

Reference	Oxide Composition /Deposition Process	Source-Drain Deposition/Channel Length	Laser Specification	Laser Annealing Conditions
This study	a-IGZO/RF sputtering	ITO sputtering/2~6 μm	355 nm (Nd:YVO_4_), nanosecond (35 ns)	Scanning at 100 mm/s, 55~280 mW @ 20 μm beam size, 100 kHz
[25]	In_2_O_3_/solution process	Al evaporation/200 μm	700 nm (Ti:sapphire), femtosecond (pulse width not reported)	No scan, 30, 60, 90 s at 97 W/cm^2^, repetition rate not reported
[26]	a-IGZO/RF sputtering	MoW sputtering/200 μm	800 nm (Ti:sapphire), femtosecond (140 fs)	No scan, 3 W, 80 MHz
[27]	Stacked IZO/solution process	Sputtering/200 μm	700 nm (Ti:sapphire), femtosecond (pulse width not reported)	No scan, 50, 100, 200 s at 3 W^2^
[28]	a-IGZO/RF sputtering	ITO sputtering/80 μm	308 nm (XeCl), nanosecond (25 ns)	Scanning mode (scan speed not reported), fluence: up to 500 mJ/cm^2^
[29]	a-IGZO/RF sputtering	Mo/Pt sputtering/10 μm	308 nm (XeCl), nanosecond (25 ns)/248 nm (KrF), pulse width not reported	Single shot, fluence: 80~110 mJ/cm^2^
[30]	a-IGZO/RF sputtering	Ti/Au evaporation/50 μm	10.6 μm CO_2_, continuous wave	Scanning at 100 mm/s, up to 400 W/cm^2^

**Table 2:** V_TH_, subthreshold swing (S.S.), mobility (*μ*), and the on/off ratio (I_on_/I_off_) for different laser powers.

Laser Power	V_TH_ [V]	S.S. [V/dec]	*μ*_n_ [cm^2^/V·s]	I_on_/I_off_
Pristine	2.48	2.00	0.84	4.06 × 10^7^
54 mW	2.76	1.77	1.20	6.79 × 10^7^
60 mW	1.19	1.81	0.77	1.88 × 10^7^
77 mW	−0.77	1.81	0.35	2.41 × 10^8^
90 mW	−1.10	1.61	0.30	4.84 × 10^8^
119 mW	−1.39	1.86	1.13	2.91 × 10^7^
155 mW	−1.88	2.28	0.32	5.42 × 10^7^
202 mW	−4.47	2.64	0.03	4.62 × 10^7^
227 mW	-	-	-	3.45 × 10^0^
253 mW	-	-	-	4.27 × 10^0^
280 mW	-	-	-	1.36 × 10^1^

## Data Availability

The data supporting the findings of this study are available from the corresponding author upon request.

## Supplementary Materials

The following supporting information can be downloaded at: https://www.mdpi.com/article/10.3390/mi15010103/s1, Figure S1: Fabrication process of an a-IGZO/ITO TFT (A) ITO sputtering and patterning (B) photolithography for lift-off (C) IGZO sputtering (D) lift-off; Figure S2: SEM images of a-IGZO TFTs after a 355 nm UV pulsed laser annealing process for laser powers of (A) 119 mW, (B) 155 mW, (C) 202 mW, (D) 227 mW, (E) 250 mW, and (F) 280 mW; Figure S3: Gate-Source leakage currents of a-IGZO TFTs after a 355 nm UV pulsed laser annealing process for laser powers of (A) 119 mW, (B) 227 mW, (C) 250 mW, and (D) 280 mW; Figure S4. X-ray photoelectron spectroscopy(XPS) results of a-IGZO thin films after a 355 nm UV pulsed laser annealing process for laser beam powers of (A) 0 (pristine), (B) 60 mW, (C) 120 mW, and (D) 280 mW.
