# Supplementary material for: 355 nm Nanosecond Ultraviolet Pulsed Laser Annealing Effects on Amorphous In-Ga-ZnO Thin Film Transistors
Source: Micromachines (Basel). 2024 Jan 5;15(1):103. doi: 10.3390/mi15010103 (PMC10818844; doi:10.3390/mi15010103)
Supplement: Supplementary file 1 [file micromachines-15-00103-s001.zip › micromachines-2785154-supplementary.pdf]

micromachines

MDPI

Article

355 nm Nanosecond Ultraviolet Pulsed Laser Annealing Effects on Amorphous In-Ga-ZnO Thin Film Transistors

Sang Yeon Park [1,†], Younggon Choi [1,†], Yong Hyeok Seo [1], Hojun Kim [2], Dong Hyun Lee [1], Phuoc Loc Truong [2], Yongmin Jeon [3], Hocheon Yoo [1], Sang Jik Kwon [1], Daeho Lee [2,*], and Eou-Sik Cho [1,*]

[1] Department of Electronics Engineering, Gachon University, 1342 Seongnam-daero, Soojung-gu, Seongnam City, Gyeonggi-do, 13120, South Korea ; tkdus9@gachon.ac.kr (S. Y. P.) ; cyg1994@gachon.ac.kr (Y. C.) ; danny99hodam@gachon.ac.kr (D. H. L.) ; hyoo@gachon.ac.kr (H. Y.) ; sjkwon@gachon.ac.kr (S. J. K.) ; es.cho@gachon.ac.kr (E.-S. C.)

[2] Department of Mechanical Engineering, Gachon University, 1342 Seongnam-daero, Soojung-gu, Seongnam City, Gyeonggi-do, 13120, South Korea ; fahenz@gachon.ac.kr (H. K.) ; 202050078@gachon.ac.kr (P. L. T.) ; dhl@gachon.ac.kr (D. L.)

[3] Department of Biomedical Engineering, Gachon University, 1342 Seongnam-daero, Soojung-gu, Seongnam City, Gyeonggi-do, 13120, South Korea; yongmin@gachon.ac.kr (Y. J.)

* Correspondence: dhl@gachon.ac.kr (D. L.) ; es.cho@gachon.ac.kr (E.-S. C.)

† These authors contributed equally to this work.

Supplementary Figures.

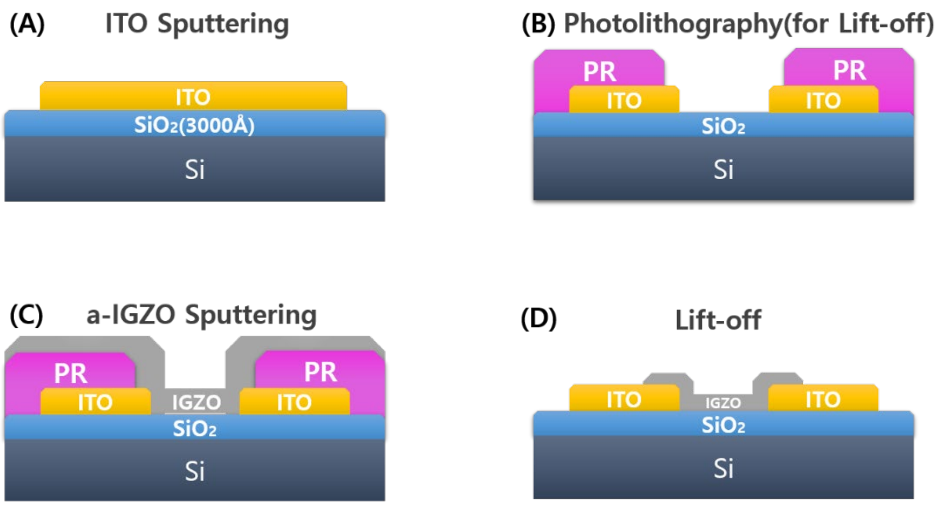

Figure S1. Fabrication process of an a-IGZO/ITO TFT (a) ITO sputtering and patterning (b) photolithography for lift-off (c) a-IGZO sputtering (d) Lift-off.

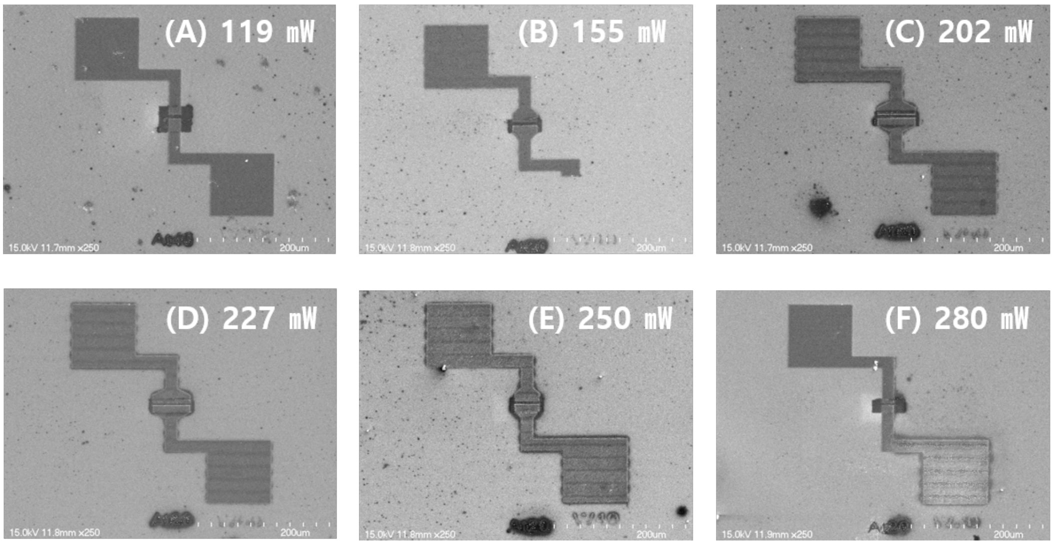

Figure S2. SEM images of a-IGZO TFTs after a 355 nm UV pulsed laser annealing process for laser powers of (a) 119 mW, (b) 155 mW, (c) 202 mW, (d) 227 mW, (e) 250 mW, and (f) 280 mW.

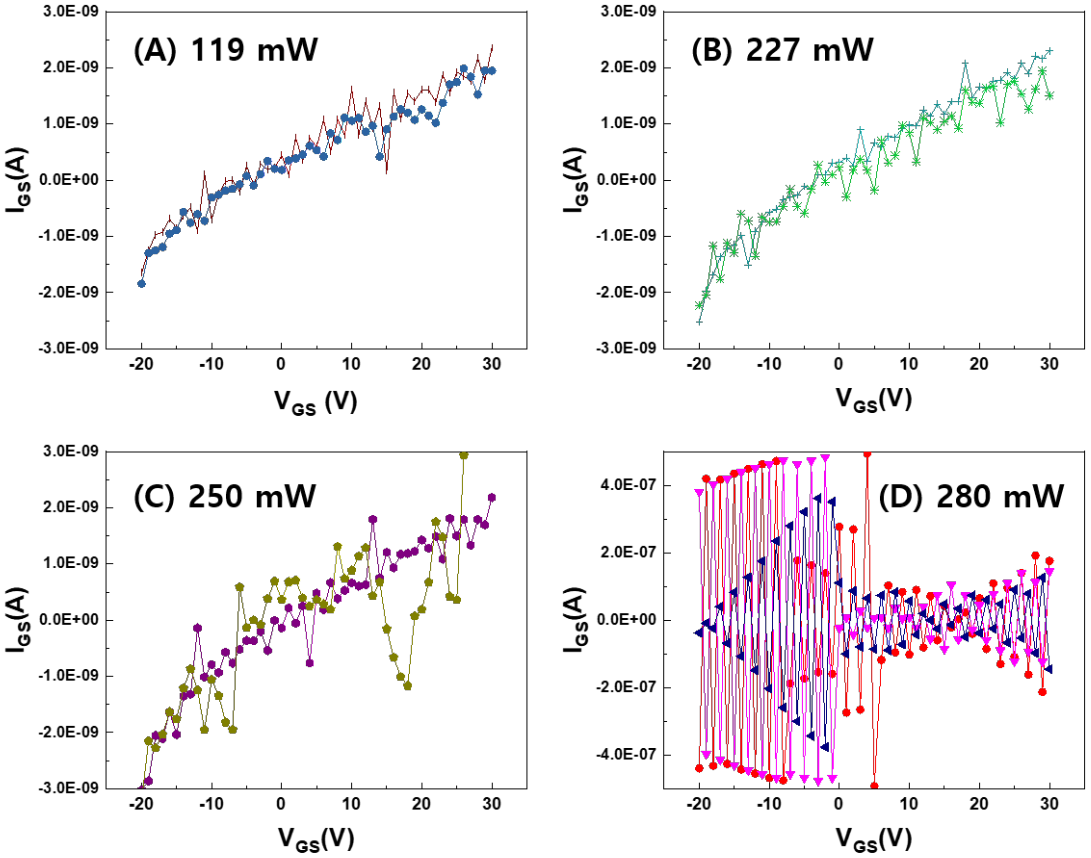

Figure S3. Gate-Source leakage currents of a-IGZO TFTs after a 355 nm UV pulsed laser annealing process for laser powers of (a) 119 mW, (b) 227 mW, (c) 250 mW, and (d) 280 mW.

• X-ray photoelectron spectroscopy (XPS) analysis of laser-annealed a-IGZO thin films with various laser powers.

The X-ray Photoelectron Spectroscopy (XPS) data for these films revealed the following trends: as the laser power increased from 0 to 120 mW, there was a noticeable increase in lattice oxygen, suggesting enhanced crystallization in the a-IGZO thin films. However, at an excessive laser power of 280 mW, the lattice oxygen content decreased. Concurrently, the presence of non-lattice oxygen – which arises from surface oxidation – escalated as the laser power increased from 0 to 280 mW. This indicates that the a-IGZO thin films undergo oxidation during the laser annealing process, and this oxidation appears to correlate with the laser power. According to the XPS results, there seems to be a complementary relationship between oxygen vacancies and lattice oxygen. As Table 1 demonstrates, mobility did not show a dependence on the laser power, suggesting that excess carriers might be too conductive to manifest distinct transfer characteristics. It is therefore conceivable that the crystallinity of the a-IGZO thin films is intricately linked to the performance of the laser-annealed a-IGZO TFTs.

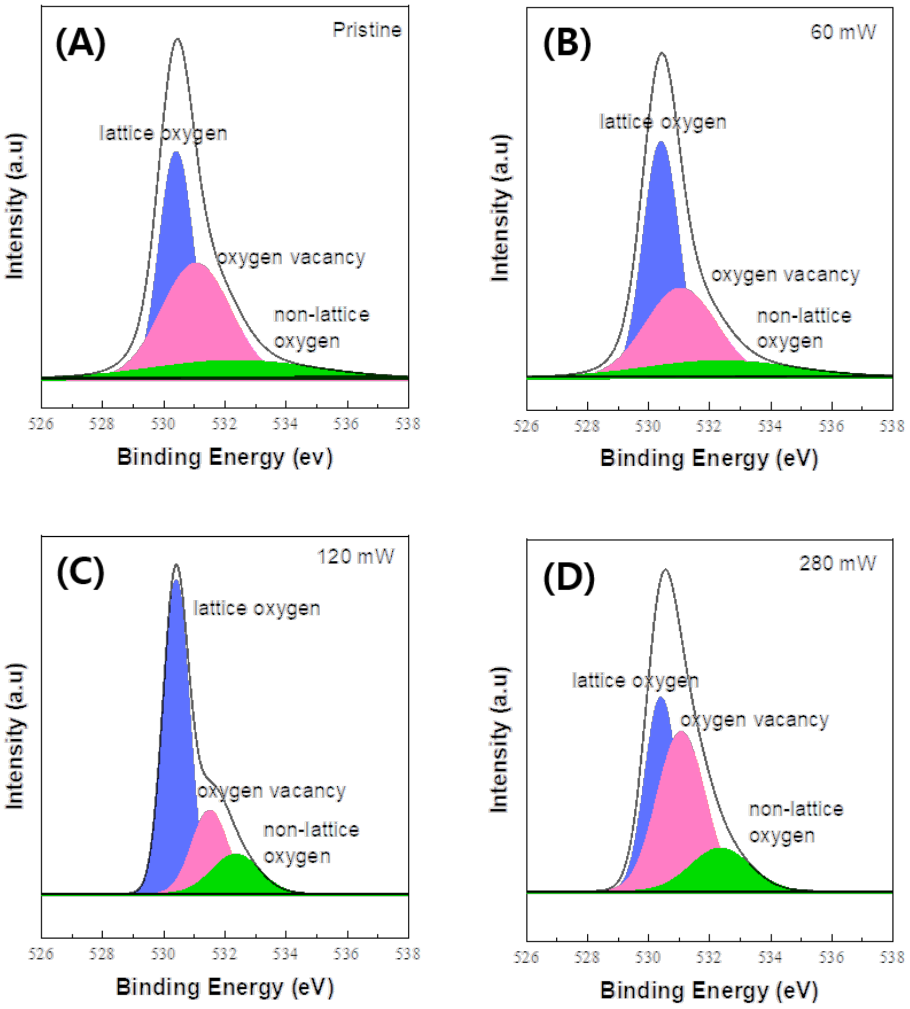

Figure S4. X-ray photoelectron spectroscopy (XPS) results of a-IGZO thin films after a 355 nm UV pulsed laser annealing process with laser powers of (a) 0 (pristine), (b) 60 mW, (c) 120 mW, and (d) 280 mW.
